# Antiplatelet Therapy in Heart Disease

**DOI:** 10.31083/RCM36522

**Published:** 2025-06-25

**Authors:** Nil Ozyuncu

**Affiliations:** ^1^Department of Cardiovascular Medicine, Ankara University School of Medicine, 06450 Ankara, Turkey

**Keywords:** antiplatelet therapy, bleeding risk, cardiovascular diseases, heart disease, ischemic risk

## Abstract

Antiplatelet therapy plays a pivotal role in the management of atherosclerotic cardiovascular diseases, providing critical protection against thrombotic complications. However, the role of antiplatelet therapy in primary prevention is limited, as an elevated risk of bleeding often offsets the potential benefits. Meanwhile, long-term antiplatelet monotherapy in secondary prevention provides clear benefits for stable patients. In the setting of acute coronary syndromes, dual antiplatelet therapy, which combines aspirin with a P2Y_12_ inhibitor, such as clopidogrel, prasugrel, or ticagrelor, has demonstrated superior efficacy over aspirin alone, with prasugrel and ticagrelor offering more rapid and potent effects. However, the increased bleeding risk associated with more intensive regimens necessitates careful assessment of both ischemic and bleeding risks, particularly in high-risk individuals. Recent advancements in stent technology and a deeper understanding of patient-specific risk profiles have led to significant advances in tailoring antiplatelet strategies. Current guidelines emphasize individualized approaches regarding the duration and intensity of the therapy. This review examines the evolution of antiplatelet treatment strategies in heart diseases, integrating evidence from pivotal studies to highlight current practices, while addressing considerations for special populations and optimal antithrombotic regimens following structural cardiac interventions. The development of novel agents, such as targeted antithrombotic therapy, and personalized therapeutic approaches continues to shape efforts to improve both efficacy and safety. Together, these advances support a more refined, patient-centered approach to antiplatelet therapy aimed at optimizing clinical outcomes in the context of a highly dynamic and evolving therapeutic landscape.

## 1. Introduction 

Cardiovascular diseases (CVD) remain a leading cause of premature mortality worldwide and contribute substantially to global healthcare expenditures. The prevalence of CVD has almost doubled, rising from 271 million cases in 1990 to 523 million in recent years [1]. Platelets play a central role in cardiovascular thrombosis by adhering to and aggregating at sites of endothelial injury, initiating thrombus formation and contributing to vascular events [2]. Antiplatelet therapy (APT), primarily consisting of aspirin (acetylsalicylic acid) and P2Y_12_ receptor inhibitors, is one of the most commonly prescribed intervention in medicine [3]. Over the past century, these agents have significantly improved clinical outcomes by preventing atherothrombotic events and reducing overall mortality [4]. Despite these advances, current APT regimens remains suboptimal, necessitating further refinement. Recent developments in APT include the introduction of P2Y_12_ inhibitors with more rapid onset of action, increased antiplatelet efficacy, and reduced interindividual variability in drug response. Additionally, novel therapeutic targets are being investigated, offering the potential for more effective and individualized treatment strategies. Innovations in stent technology and the advent of more potent antiplatelet agents have reignited discussions regarding the optimal duration of dual APT (DAPT). While a 12-month DAPT regimen has traditionally been recommended, recent studies suggest that shorter durations may minimize bleeding risks, whereas extended therapy might offer superior ischemic protection in selected high-risk populations. The increased incidence of bleeding complications associated with intensified therapy has spurred ongoing debate and highlighted the importance of individualized treatment strategies. Accordingly, there is growing emphasis on using risk stratification tools to better balance ischemic and hemorrhagic risks and tailor therapy according to each patient’s clinical profile.

Antiplatelet therapy remains a cornerstone in the prevention and management of CVD, particularly in patients with coronary artery disease (CAD), cerebrovascular events, and peripheral arterial disease. This narrative literature review aims to synthesize current evidence on the clinical application of APT, with a focus on its role in both primary and secondary prevention of cardiovascular events, management of acute coronary syndrome (ACS), and post-interventional care following structural cardiac procedures. It covers the mechanisms of antiplatelet agents, current guideline-based recommendations for mono- or dual-antiplatelet regimens, escalation and de-escalation strategies, and management of patients with concurrent indications for oral anticoagulants (OAC). Recent advances in personalized approaches and precision medicine, are also highlighted.

## 2. Mechanism of Action

**Fig. 1.**
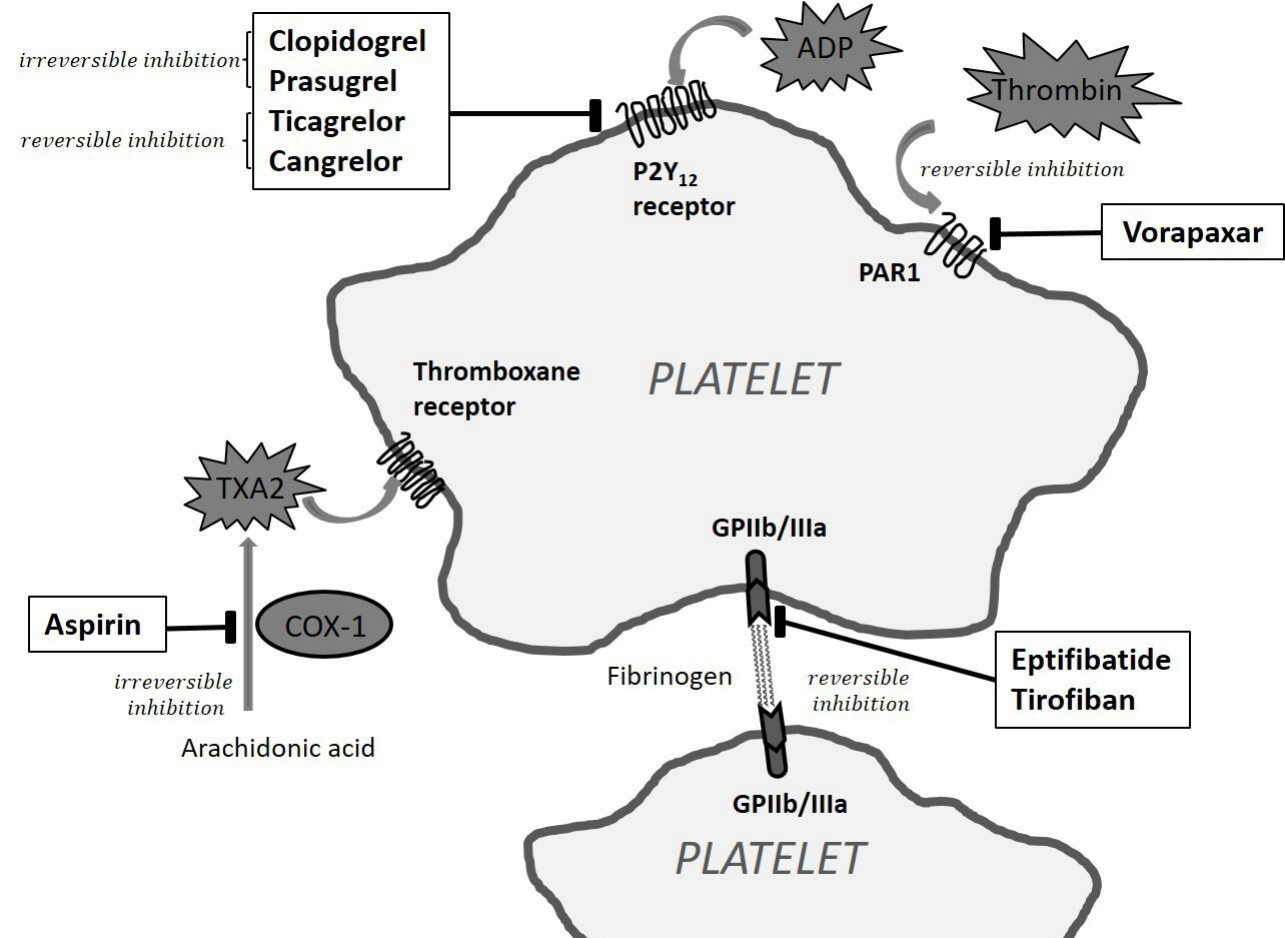
**Antiplatelet drugs primarily used for heart disease and their mechanism of action**. ADP, adenosine diphosphate; COX-1, cyclooxygenase-1 enzyme; GPIIb/IIIa, glycoprotein IIb/IIIa receptor; PAR, protease activated receptor; TXA2, thromboxane A2.

Antiplatelet drugs play a crucial role in both the treatment and prevention of atherothrombotic conditions. Platelet activation and aggregation are mediated by multiple signaling pathways, each transmitting distinct and non-redundant signals that serve as key targets for pharmacological intervention. The use of a combination APT offers additive benefits by modulating multiple pathways, thereby enhancing therapeutic efficacy. The principal antiplatelet agents in clinical practice include aspirin and P2Y_12_ receptor inhibitors, such as clopidogrel, prasugrel, ticagrelor, and cangrelor, as well as glycoprotein IIb/IIIa (GPIIb/IIIa) inhibitors. These agents exert their antithrombotic effects through diverse mechanisms, as outlined in Fig. 1.

### 2.1 Aspirin

Aspirin has remained the cornerstone of APT for over five decades and continues to be a mainstay in clinical practice. Its antiplatelet effect arises from irreversible inhibition of platelet cyclooxygenase-1 (COX-1) via acetylation of the serine residue (Ser-529), thereby preventing the access of arachidonic acid to the enzyme’s active site. This inhibition supresses the synthesis of thromboxane A2 (TXA2), a potent promoter of platelet activation and aggregation [5]. Low-dose aspirin (75–100 mg/day) achieves near-complete inhibition of TXA2 production (97–100%) with daily administration. Following aspirin discontinuation, TXA2 biosynthesis begins to recover after 24 to 48 h and returns to baseline within 7–10 days, reflecting the lifespan of circulating platelets [6]. Oral aspirin is rapidly absorbed in the stomach and small intestine, with peak plasma concentrations occuring within 30–40 min and functional platelet inhibition within 60 min. However, enteric-coated formulations may delay peak plasma concentrations to 3–4 h [7]. In some countries lysine acetylsalicylate is the only available intravenous (IV) formulation of aspirin. Compared with oral acetylsalicylic acid, IV administration results in faster and more consistent platelet inhibition [8]. Nonetheless, some evidence suggests that IV aspirin may acutely inhibit endothelial prostaglandin-mediated vasodilation, although the clinical implications of this effect remain to be elucidated [4].

Aspirin has been shown to reduce vascular mortality by approximately 15% and non-fatal vascular events by about 30%, according to a meta-analysis of over 100 randomized controlled trials [7, 9]. A significant advantage is its very low cost, making it widely accessible. Notably, its antiplatelet effect plateaus at low doses, and clinical data do not support a dose–response relationship with respect to efficacy [2, 7]. Adverse effects like gastrointestinal bleeding are dose-dependent, further supporting the use of low-dose aspirin for long-term secondary prevention [10]. In a meta-analysis of secondary prevention trials, aspirin use was associated with an increased risk of major gastrointestinal and extracranial bleeding (0.10% vs 0.07% per year, *p* < 0.0001). Despite a non-significant increase in hemorrhagic stroke, aspirin reduced total stroke incidence by 20% (2.08% vs 2.54% per year, *p* = 0.002) and coronary events by a similar margin (4.3% vs 5.3% per year, *p* < 0.0001) [11].

The term aspirin resistance refers to suboptimal platelet inhibition and is an independent predictor of future vascular events. However, “resistance” is often a misnomer, as most cases are attributed to non-adherence rather than a true pharmacodynamic failure [12, 13]. The remaining cases are largely attributed to the underlying platelet hyperreactivity predating the therapy initiation [2, 14].

### 2.2 P2Y_12_ Inhibitors

Adenosine diphosphate (ADP) is a key platelet agonist, and its binding to the P2Y_12_ receptor amplifies platelet activation and aggregation [15]. P2Y_12_ inhibitors are classified into two major groups: thienopyridines, which are prodrugs such as ticlopidine, clopidogrel, and prasugrel, and direct-acting nucleoside–nucleotide derivatives, including ticagrelor and cangrelor.

Ticlopidine, the first generation thienopyridine, was largely replaced by clopidogrel, a second-generation agent with an improved safety profile. Like ticlopidine, clopidogrel is a prodrug that requires hepatic bioactivation via cytochrome P450 (CYP450) enzymes. Its active metabolite irreversibly blocks the P2Y_12_ receptor, thereby inhibiting ADP-induced platelet aggregation [15]. Clopidogrels clinical efficacy, especially when combined with aspirin, has been demonstrated in large randomized trials in patients with ACS and those undergoing percutaneous coronary intervention (PCI) [16, 17, 18]. However, approximately 85% of the drug is inactivated by carboxylesterases before hepatic first-pass metabolism. Bioactivation requires two oxidative steps involving several CYP450 isoenzymes, particularly CYP2C19, CYP3A4, CYP2B6, and CYP2C9 (Table 1, Ref. [6, 15, 19, 20, 21, 22, 23, 24, 25]). This complex metabolism contributes to considerable interindividual variability [19]. Additionally, clopidogrel is a substrate of P-glycoprotein transporter and its pharmacokinetics may be altered by drug-drug interactions involving agents such as omeprazole or statins [26].

**Table 1.** **Pharmacological characteristics of P2Y_12_ inhibitors**.

	Clopidogrel [6, 15, 19, 21, 22, 25]	Prasugrel [6, 15, 19, 21, 22, 24, 25]	Ticagrelor [6, 15, 19, 20, 21, 22, 23, 25]	Cangrelor [6, 19, 21, 25]
Bioavailability	50%	80%	36%	100%
Reversibility	Irreversible	Irreversible	Reversible	Reversible
Half life of active metabolite	30–60 min	30–60 min	7–9 h	3–6 min
Metabolism	Activated in liver (mainly CYP2C19)	Activated in liver (mainly CYP3A4, CYP2B6)	Active drug to active metabolite in liver (CYP3A4)	Active drug*
Onset of action after loading dose	2–6 h	30 min	30 min	2 min
Frequency of administration	Once daily	Once daily	Twice daily	Intravenous infusion
Time to platelet function recovery after drug cessation	5–7 days	7–10 days	3–5 days	30–60 min
Clinical indication	CCS, PCI, ACS and ACS-PCI (in high bleeding risk patients)	ACS-PCI	ACS, ACS-PCI	Peri-PCI for bridging for the P2Y12 inhibitor naive, high ischemic risk patients

ACS, acute coronary syndrome; CCS, chronic coronary syndrome; CYP, cytochrome P450; PCI, percutaneous coronary intervention. *Not metabolised by liver, deactivated by dephosphorylation in circulation.

When added to aspirin, clopidogrel offers a 10% to 20% relative risk reduction in major vascular events in high-risk populations [6]. Nevertheless, its inconsistent P2Y_12_ inhibition has prompted the developement of more potent agents such as prasugrel and ticagrelor. Both agents exhibit a more rapid onset and increased potency, offering improved outcomes especially in the setting of ACS [27, 28]. Prasugrel is also an oral thienopyridine prodrug like clopidogrel and it requires hepatic metabolism to generate its active metabolite. Unlike clopidogrel, prasugrel undergoes initial metabolism by esterases, leading to more efficient conversion to active form and reduced variability in platelet inhibition (Table 1) [29]. Enhanced inhibition of ADP-mediated platelet activation improves protection against ischemic events, but this benefit comes with an increased risk of bleeding. The TRITON-TIMI 38 (Trial to Assess Improvement in Therapeutic Outcomes by Optimizing Platelet Inhibition with Prasugrel–Thrombolysis in Myocardial Infarction 38) trial demonstrated that in patients with ACS undergoing PCI, prasugrel significantly reduced ischemic events, including stent thrombosis, when compared to clopidogrel. However, this benefit came at the expense of increased major bleeding, including fatal hemorrhagic events [27]. Post-hoc analyses identified three subgroups; patients aged over 75 y, individuals weighing less than 60 kg, and those with history of stroke or transient ischemic attack (TIA). In the first two subgroups, the net clinical benefit was diminished, while in the latter group prasugrel use was associated with a net clinical harm [27].

Ticagrelor, a direct acting adenosine triphosphate (ATP) analogue, reversibly inhibits the P2Y_12_ receptor through non-competitive allosteric binding (Table 1) [6]. Unlike thienopyridines, ticagrelor is active in its native form and does not require metabolic activation, although its CYP3A4 mediated hepatic metabolism yields an active metabolite that also contributes to its antiplatelet effect [20]. The PLATO (Platelet Inhibition and Patient Outcomes) trial compared ticagrelor with clopidogrel in over 18,000 patients with ACS. After 12 months, ticagrelor significantly reduced composite endpoint of cardiovascular death, myocardial infarction (MI), or stroke (9.8% vs 11.7%; hazard ratio [HR]: 0.84; 95% confidence interval [CI]: 0.77–0.92; *p* < 0.001) [28]. Although ticagrelor increased the incidence of nonfatal bleeding, fatal bleeding events were not significantly higher, likely due to the reversible nature of receptor inhibition [30, 31]. Dyspnea, a common adverse effect, affects approximately 5% of patients and is a frequent reason for therapy discontinuation [32]. Cangrelor is a direct and reversible IV P2Y_12_ inhibitor with a mechanism of action similar to ticagrelor. It offers immediate platelet inhibition which resolves rapidly after discontinuation [15]. Clinical trials comparing cangrelor with clopidogrel in PCI setting demonstrated reduced periprocedural ischemic events, but with an increased incidence of minor bleeding [33, 34, 35]. Cangrelor may be considered for P2Y_12_ inhibitor naive ACS patients undergoing PCI, particularly when oral antiplatelet administration is not feasible [21].

Selatogrel (ACT-246475), a subcutaneously administered 2-phenylpyrimidine-4-carboxamide analog, is a potent, and selective P2Y_12_ inhibitor currently under investigation. It demonstrates reversible binding and a favorable safety profile in early phase trials [36]. Selatogrel Outcome Study in Suspected Acute Myocardial Infarction (SOS-AMI) trial, is evaluating its efficacy and safety in preventing all-cause mortality in ACS patients with a recent history of MI (Clinical Trials ID: NCT04957719).

### 2.3 Glycoprotein IIb/IIIa Inhibitors

Intravenous glycoprotein IIb/IIIa (GPIIb/IIIa) inhibitors function by blocking the binding of fibrinogen to the activated GPIIb/IIIa receptor (also known as α2bβ3) (Fig. 1). This inhibition prevents the formation of fibrinogen bridges between platelets, thereby effectively suppressing platelet aggregation. Abciximab, the first drug developed in this class, was withdrawn from the market in 2019 due to shifts in clinical practice and concerns regarding availability [4]. Currently, tirofiban, a non-peptide tyrosine derivative, and eptifibatide, a cyclic heptapeptide, are the main agents in use. These compounds selectively bind to the GPIIb/IIIa receptor, acting as competitive antagonists that mimick fibrinogen to prevent its interaction with the receptor [37, 38]. Due to their short plasma half-lives, administration requires an initial bolus followed by continuous infusion to maintain therapeutic levels. Evidence does not support the routine use of GPIIb/IIIa inhibitors in patients with ACS undergoing coronary angiography. Instead, their use is generally restricted to bailout situations, such as during PCI when thrombotic complications or no-reflow phenomena occur. They may also be considered in high-risk PCI cases where patients have not received pre-treatment with P2Y_12_ inhibitors [21].

### 2.4 Targeting Thrombin

The pathogenesis of ACS is primarily initiated by the rupture or erosion of the fibrous cap of an atherosclerotic plaque, which not only triggers platelet aggregation, but also exposes subendothelial tissue factor to circulating blood [39]. This exposure activates the coagulation cascade, ultimately leading to thrombin generation. Thrombin plays a critical role in converting fibrinogen into fibrin, stabilizing the thrombus, and strongly activating platelets, thereby promoting further thrombus growth through a positive feedback mechanism [40].

Vorapaxar is an oral, selective, and competitive antagonist of protease-activated receptor-1 (PAR-1), the primary thrombin receptor on human platelets (Fig. 1) [36]. In a phase III clinical trial, the addition of vorapaxar (2.5 mg once daily) to DAPT (aspirin and a P2Y_12_ inhibitor) demonstrated efficacy in secondary prevention for patients with history of atherothrombotic events, particularly MI. However, its use was associated with a significant increase in major bleeding events, including intracranial hemorrhage [41]. According to the American College of Cardiology (ACC) and the American Heart Association (AHA) Guidelines for Chronic Coronary Disease, vorapaxar may be considered as an adjunct to aspirin for reducing major adverse cardiovascular events (MACE) in post-MI patients who are at low bleeding risk and have no history of stroke, TIA, or intracranial hemorrhage (Class IIb, Level of Evidence B) [42].

Inhibition of factor Xa using rivaroxaban has also shown efficacy in reducing ischemic events in the post-ACS patients and those with established vascular disease [43, 44, 45]. Nevertheless, the increased risk of major bleeding necessitates a careful assessment of the risk-benefit ratio before initiating therapy. The role of rivaroxaban in secondary prevention, particularly in combination with APT for high ischemic risk patients, will be addressed in later sections.

Recently, factor XI has emerged as a promising therapeutic target due to its ability to modulate thrombin generation while minimally impacting hemostasis [46]. This distinction suggests that targeting factor XIa may offer effective antithrombotic protection with reduced bleeding risk, making them attractive candidates for post-ACS management [40]. Asundexian has shown >90% dose-dependent inhibition of factor XIa without an associated increase in bleeding events in patients with recent MI on DAPT. However, the study was underpowered to definitively assess efficacy [47]. Milvexian, another factor XI inhibitor, is currently undergoing clinical evaluation for its potential in preventing recurrent cardiovascular events in the post-ACS setting (Clinical Trials ID: NCT05754957).

## 3. Antiplatelet Therapy for Primary Prevention

The role of aspirin use in the primary prevention of CVD remains a subject of ongoing debate, with no unified stance among leading cardiology societies worldwide [48]. This lack of consensus largely stems from heterogeneity in study populations and conflicting evidence regarding the balance between cardiovascular benefits and bleeding risks [49, 50, 51]. Even among individuals at elevated cardiovascular risk, the net clinical benefit of aspirin appears modest and is frequently offset by an increased incidence of bleeding. A recent meta-analysis by the U.S. Preventive Services Task Force (USPSTF) concluded that low-dose aspirin significantly reduces the risk of cardiovascular events. However, this reduction did not translate into decreased cardiovascular or all-cause mortality, in line with findings from previous studies. Additionally, aspirin use was associated with a statistically significant increase in hemorrhagic complications, including both intracranial and extracranial bleeding events. Based on these findings, the USPSTF recommends that low-dose aspirin may be considered for individuals aged 40 to 59 years with a ≥10% 10-year risk of cardiovascular event, provided they are not at increased risk of bleeding [52].

The latest guidelines from the European Society of Cardiology (ESC) also provide only a weak recommendation for aspirin use in primary prevention. It is especially limited to patients with diabetes mellitus (DM) who are at high or very high cardiovascular risk (Class IIb, Level of Evidence A). For apparently healthy individuals under 70 y of age with high or very high cardiovascular risk, the ESC emphasizes the need for further research before issuing a definitive recommendation [53]. Similarly, the ACC/AHA guidelines suggest that aspirin may be considered for individuals aged 40 to 70 years who have elevated cardiovascular risk, but no increased bleeding risk (Class IIb, Level of Evidence A) [54].

Traditional cardiovascular risk assessment tools often fail to capture the complexity and heterogeneity of real-world clinical scenarios. Therefore, a more individualized approach is warranted. In addition to standard risk scores, clinicians are encouraged to evaluate patient-specific risk enhancers such as elevated lipoprotein(a) levels and coronary artery calcium (CAC) scores derived from cardiac computed tomography (CT) [55]. A recent systematic review demonstrated that among individuals with high cardiovascular risk and low bleeding risk, a CAC score of ≥100 was associated with a net clinical benefit from aspirin use in primary prevention. In contrast, among patients with an elevated risk of bleeding, aspirin use was linked to net harm, irrespective of CAC score or overall cardiovascular risk [56].

## 4. Antiplatelet Therapy for Secondary Prevention

Secondary prevention in cardiology focuses on reducing the risk of recurrent cardiovascular events in individuals with a history of CVD. The latest European guidelines classify patients with CAD into two categories based on clinical presentation: ACS and chronic coronary syndrome (CCS) [10, 21]. This classification enables clinicians to adapt management strategies according to the patient’s clinical status. Notably, patients may transition between ACS and CCS phases throughout the course of their disease.

### 4.1 Chronic Coronary Syndrome

Chronic coronary syndrome includes a broad range of patients from asymptomatic individuals with incidentally detected CAD on imaging to those recovering from ACS or undergoing follow-up after revascularization. It also encompasses patients with anginal symptoms or ischemia attributable to vasomotor dysfunction or microvascular disease, even in the absence of significant epicardial stenosis [10]. In this context, low-dose aspirin remains the cornerstone of APT [10].

Although APT administered in the initial years post-ACS technically falls under the CCS phase, it will be addressed in the ACS section to provide a cohesive overview of treatment across the ACS timeline.

#### 4.1.1 Asymptomatic Individuals With Evidence of Coronary Artery Disease on Imaging

The incidental detection of epicardial CAD during routine imaging, such as coronary CT angiography or carotid ultrasonography, has become increasingly common. Imaging evidence of significant atherosclerotic plaque is indicative of very high cardiovascular risk [57]. For patients with significant CAD on imaging, lifelong aspirin therapy is recommended for secondary prevention [10, 42]. DAPT with clopidogrel is not indicated in this group [10, 58]. However, clopidogrel is recommended as an alternative to aspirin for secondary prevention in cases of aspirin intolerance [53].

The clinical implications of detecting non-obstructive plaques or elevated CAC scores remain uncertain. While such findings are associated with an increased risk of future atherosclerotic events, current guidelines provide limited direction and evidence from meta-analyses remains inconclusive [11, 48]. In these cases management is typically guided by primary prevention strategies using risk scores such as the Atherosclerotic Cardiovascular Disease (ASCVD) Risk Calculator or Systematic Coronary Risk Evaluation 2 (SCORE2) [42, 53]. APT is not recommended for individuals with low or moderate CVD risk [53]. Risk assessment should be further refined by using risk modifiers, such as CAC scoring and carotid ultrasonography.

Bleeding risk should also be evaluated to ensure an individualized risk-benefit based approach to APT initiation. The ongoing Efficacy and Safety of Aspirin in Patients With Chronic Coronary Syndrome Without Revascularization trial (ASA-IN) (NCT05347069) aims to generate evidence on the efficacy and safety of aspirin in patients without a prior MI or revascularization, potentially informing future clinical recommendations [48].

#### 4.1.2 Antiplatelet Therapy in Symptomatic Chronic Coronary Syndrome Patients

For patients with significant CAD, but without a history of MI or PCI, lifelong low-dose aspirin therapy is recommended (Class I, Level of Evidence B). In individuals with a history of MI or PCI, the recommendation remains Class I, but with a higher Level of Evidence (A) [10]. The same recommendation applies to those who have undergone coronary artery bypass grafting (CABG). In selected patients with a low risk of bleeding but a high risk of thrombotic graft occlusion, DAPT may be considered in the early postoperative period following CABG [10].

For CCS patients undergoing elective PCI, the standard antiplatelet regimen involves DAPT with aspirin and clopidogrel for up to 6 months. The recommended loading doses are 150–300 mg of aspirin and 600 mg of clopidogrel (a 300 mg dose should be considered if fibrinolytic therapy was administered), followed by standard maintenance doses [10, 59]. In patients undergoing high-thrombotic-risk procedures such as complex left main interventions, two-stent bifurcations, suboptimal stent deployment, prior stent thrombosis, or those with CYP450 polymorphisms prasugrel or ticagrelor may be considered as alternatives to clopidogrel. In such cases, intensified DAPT is typically recommended during the first month and may be extended up to 3–6 months depending on thrombotic risk [10]. In patients initially managed with ticagrelor-based DAPT following PCI, monotherapy with ticagrelor may be considered after the initial DAPT phase. This approach may serve as an alternative to prolonged DAPT or switching to another single antiplatelet agent and is particularly suitable for patients with high ischemic risk and low bleeding risk profiles [10, 60, 61].

Although both ischemic and bleeding risks peak during the periprocedural phase, their trajectories differ over time. While bleeding risk remains relatively stable, ischemic risk decreases markedly within 1 to 3 months post-PCI, although this trend may vary based on individual clinical presentations and procedural complexity [62]. These evolving risk profiles have prompted growing interest in tailoring APT to individual patient characteristics to optimize clinical outcomes through personalized treatment strategies. When both bleeding and ischemic risks are present, observational studies suggest prioritizing bleeding risk in determining the duration of DAPT [63]. Current clinical guidelines support this approach, recommending that bleeding risk should primarily guide decisions on the appropriate duration [21].

In patients identified as having a high bleeding risk (HBR) but not an elevated ischemic risk, guidelines recommend discontinuing DAPT within 1 to 3 months following elective PCI, followed by continuation of single APT (SAPT). For patients without high bleeding or ischemic risk, early DAPT discontinuation within this same period is categorized as an option that “may be considered” [10]. Numerous clinical trials have evaluated the safety and efficacy of abbreviated DAPT followed by monotherapy, most commonly with clopidogrel, compared to the standard 12-month regimen. The SMART-CHOICE trial demonstrated that 3 months of DAPT followed by P2Y_12_ inhibitor monotherapy was noninferior to 12 months of DAPT in preventing MACE, while also significantly reducing major bleeding [64]. Similarly, the STOPDAPT-2 trial, which included both acute and CCS patients, found that a 1-month DAPT regimen followed by clopidogrel monotherapy was noninferior to the traditional 12-month DAPT strategy in preventing cardiovascular events and was associated with a lower incidence of bleeding [65]. Reflecting these findings, the 2023 ACC/AHA Guideline for CCS endorses early transition to P2Y_12_ inhibitor monotherapy after 1 to 3 months of DAPT as a reasonable evidence-supported strategy [42].

Aspirin-free strategies for long-term secondary prevention in patients with prior MI or revascularization have also been investigated. The ESC Guideline for CCS recommends clopidogrel 75 mg daily as a safe and effective alternative to aspirin monotherapy in these patients [10, 66, 67, 68, 69]. The HOST-EXAM Extended study provided long-term insights, showing that clopidogrel monotherapy was superior to aspirin monotherapy in patients who remained event-free for 12 ± 6 months following PCI. Over more than 5 year of follow-up, clopidogrel was associated with significantly lower rates of composite net clinical outcomes, including MACE and bleeding events [70]. Further supporting evidence comes from the PANTHER collaboration, which analyzed data from 24,325 patients with a history of MI or revascularization. The study found that P2Y_12_ inhibitor monotherapy, predominantly clopidogrel, was associated with significantly lower rates of MACE over a 2-year follow-up period compared with aspirin monotherapy. This benefit was largely attributed to a reduced incidence of MI, with a number needed to treat of 136. Importantly, there was no significant difference in major bleeding rates between the two monotherapy strategies, suggesting that clopidogrel may be both safer and more effective than aspirin in specific patient populations [66]. These findings challenge the traditional preference for aspirin as the first-line agent for SAPT [71]. The recent SMART-CHOICE 3 trial revealed that in patients at high risk of recurrent ischemic events, clopidogrel monotherapy following standard DAPT after PCI was associated with a significantly lower cumulative incidence of MACE, without an increased risk of bleeding [72]. Despite its clinical advantages clopidogrel may not offer the same cost-effectiveness as aspirin. Aspirin remains the more economically viable option for lifelong secondary prevention. Given the variability in drug pricing, healthcare infrastructure, and reimbursement systems across countries, further health economic analyses are warranted to assess the long-term cost-effectiveness of clopidogrel-based strategies across diverse clinical and socioeconomic settings.

#### 4.1.3 Antiplatelet Therapy in Patients With an Oral Anticoagulant Indication

Patients with CAD may have accompanying atrial fibrillation (AF) or have coexisting conditions necessitating OAC therapy. Approximately 20% of patients with AF require PCI, which introduces the need for combined treatment with OAC (preferably a direct OAC [DOAC] over a vitamin K antagonist [VKA]) and DAPT. This combination, known as triple antithrombotic therapy (TAT), significantly elevates the risk of bleeding [73]. VKAs are associated with a less favorable safety profile, particularly in terms of major and fatal bleeding [48]. Therefore, current guidelines recommend DOACs over VKAs unless contraindicated [10]. Given the well-established association between major bleeding and increased mortality, minimizing bleeding risk is a key therapeutic objective. In CCS patients who require anticoagulation but have no recent history of MI or PCI, OAC monotherapy is preferred. This strategy strikes a balance between minimizing bleeding risk and preventing cardioembolic events.

In patients undergoing PCI, APT must be carefully managed to balance ischemic protection with bleeding risk. In such cases, low-dose aspirin is added to OAC and clopidogrel as part of initial regimen. Following an uncomplicated PCI, early discontinuation of aspirin (≤1 week post-procedure) is advised, with continued use of OAC and clopidogrel for up to 6 months in patients with low ischemic risk, and up to 12 months in those with high ischemic risk. After this period, OAC monotherapy should be resumed for long-term management [10, 74, 75]. In patients with a higher ischemic risk, continuation of aspirin for up to 1 month post-PCI should be considered if the ischemic risk outweigh the bleeding risk. Notably, prasugrel and ticagrelor are not recommended for use as part of TAT [10].

### 4.2 Acute Coronary Syndrome

Patients with ACS are categorized based on initial electrocardiogram findings to guide immediate and long-term treatment. The two main categories are ST-segment elevation MI (STEMI) and non-STEMI (NSTEMI), including unstable angina pectoris (UAP). According to current international guidelines, 12 months of DAPT remains the cornerstone of management in ACS, irrespective of whether patients undergo medical treatment or PCI [21].

#### 4.2.1 Antiplatelet Therapy in the Acute Phase 

Aspirin is recommended for all eligible patients in the acute phase, unless contraindicated. An initial loading dose of 150–300 mg should be administered, followed by low dose maintenance. In addition to aspirin, a P2Y_12_ inhibitor is recommended and should be initiated with a loading dose and maintained for 12 months, provided there is no high risk of bleeding. In patients undergoing CABG who temporarily discontinue their P2Y_12_ inhibitor, the 12-month DAPT course should be resumed postoperatively.

For ACS patients undergoing PCI, prasugrel and ticagrelor are preferred over clopidogrel [21]. Ticagrelor is also recommended in medically managed ACS patients, whereas prasugrel is not approved for this group. Among patients scheduled for invasive therapy, prasugrel has been shown to significantly reduce cardiovascular event risk compared with ticagrelor [76]. Accordingly, the 2023 ESC guideline for ACS gives a Class IIa recommendation (Level of Evidence B) to prasugrel over ticagrelor in patients proceeding to PCI [21]. Clopidogrel should be used when the other P2Y_12_ inhibitors are contraindicated, unavailable, or not tolerated [21].

Pre-treatment with a P2Y_12_ inhibitor, administering a loading dose before coronary angiography, is intended to ensure early platelet inhibition. However, this strategy remains controversial due to lack of robust evidence from large randomized trials. Pretreatment may unnecessarily increase bleeding risk in patients who ultimately do not require PCI or who need emergent CABG. Patients requiring urgent or early CABG may face increased risk of adverse outcomes due to the need to delay surgery until the antiplatelet effect has sufficiently diminished. For all ACS patients undergoing PCI, who have not received prior P2Y_12_ inhibition, a loading dose is recommended at the time of the procedure [21]. In STEMI patients, pre-treatment may be considered due to the typically high ischemic risk. However, in NSTEMI patients undergoing early invasive management, routine pre-treatment is not recommended unless the coronary anatomy is already known. If an invasive strategy is expected to be delayed (>24 h), pre-treatment with a P2Y_12_ inhibitor may be considered [21, 77, 78].

Intravenous. antiplatelet agents such as cangrelor and GPIIb/IIIa inhibitors were discussed in the previous section on mechanisms of action. At present, there is insufficient evidence to support their routine use in ACS patients undergoing coronary angiography. Their use should be limited to selected cases and evaluated individually [21].

#### 4.2.2 Long-Term Antiplatelet Therapy After Acute Coronary Syndrome and Tailoring the Treatment

Following an ACS, the standard recommendation is to administer DAPT, typically comprising aspirin and a potent P2Y_12_ receptor inhibitor for 12 months, regardless of the treatment strategy employed, provided there are no contraindications. In older patients with ACS, a DAPT regimen consisting of aspirin and clopidogrel may also be clinically beneficial [79].

For nearly two decades, this 12-month DAPT duration has been strongly endorsed by both American and European guidelines for the management of ACS [21, 80]. However, with the advent of stent technologies and more potent antiplatelet agents, the optimal duration of DAPT has become a subject of ongoing debate. Trials focused on DAPT duration have not demonstrated a consistent net benefit for the 12-month regimen, either due to higher bleeding rates associated with longer therapy or insufficient ischemic protection with shorter regimens [81]. In clinical practice, the duration and intensity of DAPT may be adjusted, either shortened (<12 months), extended (>12 months), or modified (e.g., switching agents or de-escalating therapy), depending on individual patient’s ischemic and bleeding risk profile [21]. Personalizing therapy is now considered essential for optimizing the net clinical benefit.

4.2.2.1 Balancing Bleeding and Ischemic Risk

The conventional 12-month DAPT regimen offers substantial ischemic protection but increases the risk of bleeding, which can have serious prognostic implications. As awareness grows regarding the adverse outcomes associated with bleeding, more personalized strategies are being adopted to better balance ischemic benefits and bleeding risks. In clinical trials assessing DAPT following PCI in ACS patients, major bleeding during the first year has been reported in 1% to 10% of cases [82, 83]. Importantly, major bleeding is linked to a nearly threefold increase in mortality within the first year and a fivefold increase in the risk of death or MI within the first 30 days [84, 85].

Bleeding risk is influenced by both modifiable and non-modifiable factors. Several clinical scoring systems have been developed to quantify bleeding risk in patients undergoing APT [86, 87]. Among them, the PRECISE-DAPT score is tailored specifically to predict bleeding events after PCI [86]. The Academic Research Consortium for High Bleeding Risk (ARC-HBR) has also developed a consensus-based definition to identify high risk patients, comprising 20 clinical criteria categorized as major or minor [88]. More recently, the PRECISE-HBR score, a validated bleeding risk model was introduced, combining elements of the PRECISE-DAPT score and ARC-HBR criteria. It simplified the risk assessment by combining seven key clinical variables into a user-friendly format aiming to predict post-PCI bleeding events. Compared to existing models, the PRECISE-HBR score offers a modest yet meaningful improvement in predictive accuracy and may enhance clinical decision-making in tailoring APT [89]. Despite their validation in real-world settings, existing risk models fail to account for factors such as frailty, low body weight, or heart failure, potentially leading to underestimation of bleeding risk in certain populations. Hence, clinical judgement remains crucial alongside the use of risk stratification tools [82].

A “bleeding-focused” approach is increasingly influencing post-PCI antiplatelet strategies. However, data on the role of IV antiplatelet agents, such as cangrelor, in predicting or preventing periprocedural bleeding are still limited. The Intravenous Cangrelor in High-Bleeding Risk Patients Undergoing PCI (ICARUS) study was a retrospective observational study that assessed real-world use of cangrelor and developed a bleeding risk prediction model. The validated ICARUS score, based on three variables: age, acute clinical presentation, and femoral access, offers a simple yet effective method for identifying patients at high risk of periprocedural bleeding. In such patients, the use of cangrelor should be approached with caution and accompanied by close postprocedural monitoring [90].

Patients with ACS undergoing PCI remain at risk of subsequent ischemic events, especially in the early period (approximately 5% within the first year) [91]. Both patient-specific and procedural factors contribute to this risk [82]. Notably, many clinical characteristics that predict ischemic events also correlate with bleeding risk, particularly among elderly patients. The DAPT score can help differentiate patients more likely to benefit from extended DAPT regimens, while minimizing bleeding risk [92]. A systematic review and meta-analysis involving over 88,000 patients undergoing elective or ACS-related PCI confirmed the ability of DAPT score to effectively balance ischemic and bleeding risks [93]. According to clinical guidelines, bleeding risk should take precedence when determining DAPT duration [21].

APT can be escalated (increased intensity via drug type, dose or number of agents) or de-escalated (reduced intensity or therapy simplification) to tailor treatment to the patient’s clinical profile [94].

4.2.2.2 De-Escalation Strategies

De-escalation strategies are employed when the risk of bleeding outweighs the risk of thrombotic events. These strategies may involve switching to a less potent antiplatelet agent, lowering the dose, or discontinuing one component of DAPT. In most cases de-escalation focuses on modifying the P2Y_12_ inhibitor. Transitioning from DAPT to P2Y₁₂ inhibitor monotherapy is also considered a form of de-escalation [94].

The latest ESC guidelines for ACS recommend alternatives to the conventional 12-month DAPT regimen, including shortening the DAPT duration to 1 or 3–6 months, based on individual bleeding and ischemic risks. Another option is to de-escalate from a prasugrel- or ticagrelor-based regimen to one centered on clopidogrel. However, much of the supporting evidence for these alternatives is derived from trials primarily powered to detect bleeding outcomes, with most designed to show non-inferiority rather than superiority [21]. Therefore, such strategies should be considered as alternative approaches and not yet replace the standard 12-month course of DAPT. Practically, they are best reserved for selected patients, particularly those at HBR. De-escalation within the first 30 days post-ACS is not recommended; however, beyond 30 days, de-escalation of DAPT and switching to monotherapy may be considered to minimize bleeding risk [21, 95, 96].

Clopidogrel monotherapy in place of aspirin is gaining broader acceptance, supported by robust trial data [69, 70, 97, 98]. The STOPDAPT-2 ACS extension trial randomized 3008 ACS patients undergoing PCI to receive either 1–2 months of DAPT followed by clopidogrel monotherapy or conventional 12-month DAPT (aspirin plus clopidogrel). The study found that clopidogrel monotherapy failed to demonstrate non-inferiority for net clinical benefit compared to conventional DAPT and was associated with an increased rate of MI [99]. Therefore, clopidogrel monotherapy 3 months post-PCI may be the most appropriate de-escalation strategy for patients whose bleeding risk outweigh their ischemic risk. Additionally, de-escalating the P2Y_12_ inhibitor, switching from prasugrel or ticagrelor to clopidogrel, may serve as an effective strategy to reduce bleeding [21, 96, 100, 101].

De-escalation may be implemented empirically (unguided), relying solely on clinical judgment, or can be guided by platelet function testing (PFT) or CYP2C19 genotyping, based on the patient risk profiles and resource availability. Recent meta-analyses have shown that both guided and unguided de-escalation reduce bleeding risk without increasing ischemic events [102, 103]. Both the recent ESC and ACC/AHA guidelines for ACS do not give a specific recommendation for guided DAPT abbreviation [21, 96].

Alternatives to routine 12-month DAPT have been extensively studied. The rationale for de-escalation stems from the observation that ischemic risk is highest in the early months post-ACS, whereas bleeding risk remains constant or may eventually outweigh ischemic risk by time [82, 104]. The TICO randomized trial (the effect of ticagrelor monotherapy vs ticagrelor with aspirin on major bleeding and cardiovascular events in patients with acute coronary syndrome), which assessed ticagrelor monotherapy after 3 months of DAPT versus continued ticagrelor-based DAPT for 12 months, found a modest yet statistically significant reduction in the composite endpoint of major bleeding and cardiovascular events at 1 year (HR: 0.56; 95% CI: 0.34 to 0.91; *p* = 0.02 for major bleeding, HR: 0.66; 95% CI: 0.48 to 0.92; *p* = 0.01 for net adverse clinical events at first year) [105]. According to ESC guidelines, single APT, preferably as P2Y_12_ inhibitor monotherapy, should be considered in patients who remain event-free after 3–6 months of DAPT and who are not at high ischemic risk [21]. It should be noted that this recommendation is not exclusive for the HBR patients, it is an alternative DAPT strategy with a Class IIa (Level of Evidence A) recommendation for event free population without high ischemic risk. The 2025 ACC/AHA guideline of ACS support transitioning to ticagrelor monotherapy ≥1 month post-PCI in ACS patients who have tolerated DAPT well, with a Class I recommendation (Level of Evidence A) based on its ability to reduce bleeding without compromising ischemic protection [96].

Two unguided DAPT de-escalation strategies have emerged as promising for patients without high ischemic risk: (a) switching from potent P2Y_12_ inhibitor to clopidogrel and (b) discontinuing aspirin and continuing with P2Y_12_ monotherapy at 3–6 months post-ACS [82, 106, 107]. Although these strategies have not been directly compared in head-to-head trials, they should be assessed in eligible patients to optimize outcomes [82].

4.2.2.3 Escalation Strategies

Escalation strategies are employed to reduce ischemic complications by intensifying platelet inhibition when the risk of ischemic events outweighs the potential for bleeding. This can be achieved by switching to a more potent antiplatelet agent (particularly in elective PCI patients following high-risk procedures or in those with recurrent events), increasing the current drug dosage, or adding an additional antiplatelet agent [94].

Extending the duration of DAPT beyond the standard post-ACS regimen also constitutes an escalation of APT. Prolonged DAPT with P2Y_12_ inhibitors (mostly clopidogrel and ticagrelor) for up to 30–36 months has been shown to reduce primary ischemic end points in patients with high ischemic risk and without HBR, although this benefit comes with an increased bleeding risk [92, 108]. Furthermore, in patients with CCS and high ischemic risk, adding low-dose rivaroxaban (2.5 mg twice daily) to aspirin monotherapy has demonstrated efficacy in reducing ischemic events, albeit with a concomitant increase in bleeding risk [45]. Based on current clinical evidence, in patients without HBR, the addition of a second antithrombotic agent to aspirin for extended long-term secondary prevention should be considered in those with high ischemic risk and may be considered in those with moderate ischemic risk [21].

Importantly, the presence of HBR should remain the primary determinant when deciding on the duration and intensity of APT, regardless of the patient’s ischemic risk [109]. Additionally, current bleeding risk scoring tools may underestimate the bleeding potential in certain subpopulations. HBR should be assumed in patients with a history of major bleeding and those presenting with anemia [86]. For such patients, monotherapy with a P2Y_12_ inhibitor may be a reasonable option, as supported by emerging clinical data [109].

#### 4.2.3 Antiplatelet Therapy in Patients With Oral Anti-Coagulant Indication 

In patients with an indication for OAC and no contraindications, current evidence favors the use DOACs over VKAs, due to their reduced bleeding risk. According to the ESC guidelines, the recommended strategy involves dual antithrombotic therapy consisting of a standard dose DOAC for stroke prevention and a single antiplatelet agent, preferably clopidogrel. This regimen is advised for up to 12 months following an initial period up to 1 week of TAT, which includes a DOAC, aspirin and clopidogrel. The use of prasugrel or ticagrelor as part of TAT is not recommended [21]. In patients treated with VKA close monitoring of anticoagulation intensity is essential.

For patients with an indication for OAC and a high ischemic risk that outweighs the bleeding risk, extending the use of aspirin and clopidogrel beyond 1 week, up to 1 month should be considered. Additionally, in selected patients, discontinuation of APT at 6 months while continuing OAC may be appropriate to mitigate bleeding risk. In medically managed patients who require OAC, a SAPT in combination with OAC should be considered for up to 1 year [21].

## 5. Considerations in Specific Patient Populations

### 5.1 Elderly

Advanced age independently increases cardiovascular risk and is also associated with a progressive rise in bleeding risk with APT. A sub-study of the PLATO trial comparing ticagrelor and clopidogrel as part of DAPT found that the benefits of ticagrelor were consistent across age groups [110]. However, the recently published POPular AGE trial challenged this, showing that in elderly patients, clopidogrel provided comparable antithrombotic efficacy with more potent agents while resulting in fewer bleeding events [79].

In individuals aged over 75 years, the incidence of upper gastrointestinal bleeding associated with aspirin increases from approximately 0.5 to 1 per 100 patient-years. Aspirin for secondary prevention appears more beneficial in patients aged 64–74 years, than those aged 50–59 years, suggesting that advanced age alone should not preclude secondary prevention strategies [111]. Patients presenting with upper gastrointestinal pain or a history of ulcers, particularly those aged ≥65 years receiving DAPT, should receive gastroprotection with a proton pump inhibitor [19]. In cases of ACS, current guidelines suggest that clopidogrel may be considered the P2Y_12_ inhibitor of choice in older patients, particularly those with HBR [21]. APT in the elderly should be individualized based on a comprehensive evaluation of ischemic and bleeding risks, life expectancy, comorbidities, frailty, cognitive and functional status, and potential need for non-cardiac surgery.

### 5.2 Kidney Disease

Moderate to-severe chronic kidney disease (CKD; stages III–V) affects over 30% of patients with ACS. APT strategy in this population follows recommendations for individuals at very high cardiovascular risk category. However, clinical decision making is often limited by the exclusion of patients with end-stage renal disease (ESRD) or those on dialysis from randomized trials [112]. These patients typically receive fewer interventional and pharmacological therapies and experience worse outcomes, which are attributable to a higher burden of comorbidities and increased risk of in-hospital complications, including major bleeding events [113]. Antiplatelet therapy choice and DAPT duration should be carefully tailored based on renal function and the patient’s ischemic and bleeding risk profiles. In patients with ESRD (eGFR ≤15 mL/min/1.73 m^2^), clopidogrel remains the only P2Y_12_ inhibitor with limited supporting evidence. The use of prasugrel and ticagrelor is not recommended in this group [21].

### 5.3 Patients With Diabetes

Given the prothrombotic nature of diabetes, antiplatelet prophylaxis has traditionally been used for primary prevention. However, evidence suggests that aspirin provides no net benefit for primary prevention in patients with diabetes without established CVD [11]. The latest ESC guidelines for CVD prevention recommend that low dose aspirin may be considered for primary prevention in patients with diabetes with high or very high CVD risk [53].

Diabetes is a well-established risk factor for ischemic events. Notably, the TRITON-TIMI 38 trial demonstrated a greater relative reduction in ischemic events among patients with diabetes receiving DAPT post-PCI, compared with nondiabetic individuals (17% versus 12.2%, HR: 0.70; 95% CI: 0.58–0.85; *p* < 0.001) [27]. Although current evidence supports the use of similar APT regimens in both diabetic and nondiabetic patients, patients with diabetes may derive greater benefit from escalated APT strategies, reinforcing the need for individualized risk-based therapy.

### 5.4 Patients With Extreme Body Weights

Underweight patients are predisposed to bleeding complications. In individuals weighing <60 kg, prasugrel should either be avoided or administered at a reduced dose of 5 mg once daily [114]. Findings from the HOST-EXAM trial showed a significantly higher risk of major bleeding in underweight patients during the chronic phase of antiplatelet monotherapy following PCI compared with those of normal weight (HR: 4.140; 95% CI: 1.704–10.059; *p* = 0.002) [115].

Conversely, obesity may alter drug pharmacokinetics and pharmacodynamics, while its associated inflammatory state may increase platelet reactivity and reduce APT efficacy. Obesity has been linked to diminished aspirin response due to significant interpatient variability in drug effects [116]. The ESC recently released a clinical consensus document addressing the challenges and considerations for antithrombotic therapy across different body mass categories [117].

## 6. Precision Medicine and Personalized Approaches

Personalized therapy marks a paradigm shift in APT, emphasizing the individualization of treatment based on patient-specific characteristics, genetic predispositions, and underlying disease mechanisms. Clinicians now have access to advanced methods for evaluating ischemic and bleeding risk beyond conventional scoring systems, such as PFT and genetic profiling. Despite their potential, these strategies are not routinely integrated into clinical practice. Current guidelines recommend using risk stratification selectively to tailor the duration and composition of APT [10, 21].

Clopidogrel and prasugrel are prodrugs that require hepatic biotransformation via CYP450 enzymes to produce their active metabolites. Genetic polymorphisms, particularly loss-of-function mutations in the *CYP2C19* gene, can impair the activation of clopidogrel, resulting in reduced antiplatelet efficacy [118]. This diminished conversion contributes to high on-treatment platelet reactivity (HPR), a condition frequently observed in clopidogrel treated patients (reported in approximately 42%, with a range of 7%–75%) and considerably less common with prasugrel [119, 120]. Although genetic and PFT hold promise for optimizing individualized APT, the routine implementation of guided de-escalation strategies remains limited by logistical and cost-effectiveness constraints. In this context, unguided de-escalation approaches may offer a more practical, accessible, and economically viable alternative for contemporary antiplatelet management [121].

Platelet function testing-guided escalation of P2Y_12_ inhibitor therapy may be considered in select cases where achieving optimal platelet inhibition is essential and bleeding risk is deemed acceptable. These include patients undergoing left main stenting, PCI of the last patent vessel, or those with a history of stent thrombosis [122]. The recently developed ABCD-GENE score incorporates four clinical variables; age, body mass index, CKD, and diabetes mellitus, alongside *CYP2C19* genotype data [123]. This composite tool effectively identifies patients with HPR while on clopidogrel. Escalation of APT may be considered in patients with a high ABCD-GENE score, although prospective validation is required to confirm its clinical utility [118].

While current guidelines do not recommend routine use of genotyping or PFT they acknowledge that in patients undergoing high-risk PCI who are known carriers of a *CYP2C19* loss-of-function allele, substitution of clopidogrel with ticagrelor or prasugrel is a reasonable strategy [10, 124]. Further randomized trials are needed to clearly define which patient populations benefit most from guided versus unguided approaches, particularly in balancing ischemic protection with bleeding risk. Importantly, recent evidence supports the safety and efficacy of unguided de-escalation strategies. These approaches may provide practical and economic advantages by obviating the need for additional testing or follow-up visits, thereby enhancing accessibility and ease of implementation in real-world settings [125].

## 7. Antiplatelet Therapy After Catheter-Based Structural Cardiac Interventions

The ongoing evolution of catheter-based interventional cardiology has significantly broadened the therapeutic landscape for managing structural heart diseases. These advancements not only offer less invasive alternatives to surgery but also contribute to faster recovery times and lower healthcare costs. Notably, transcatheter aortic valve implantation (TAVI) has become a standard therapy for severe aortic stenosis, while transcatheter edge-to-edge repair (TEER) is increasingly used for the treatment of severe mitral regurgitation (MR) and tricuspid regurgitation (TR). In addition to valvular interventions, catheter-based techniques are employed to address other structural heart abnormalities, such as closure of atrial septal defects (ASDs), patent foramen ovale (PFO), and ventricular septal defects (VSDs). Left atrial appendage (LAA) occlusion has also emerged as an alternative stroke prevention strategy for patients with AF who are unable to tolerate or have contraindications to long-term OAC.

Following these structural interventions, antithrombotic therapy is essential to prevent thrombus formation, minimize embolism and ischemic complications, and ensure favorable long-term outcomes. The choice and duration of antithrombotic therapy are guided by the type of procedure performed and patient-specific factors. Commonly used agents include APT (e.g., aspirin and/or clopidogrel), VKAs, and DOACs [126]. Current guidelines recommend that patients with an established indication for DOAC or VKA should continue their anticoagulant regimen [48].

### 7.1 Valvular Interventions

#### 7.1.1 TAVI

Evidence supports a 3-month antithrombotic strategy following TAVI, coinciding with the period of neointimal coverage and endothelialization [127, 128]. Despite several randomized trials, current recommendations for post-TAVI antithrombotic therapy are still largely based on expert consensus rather than definitive clinical evidence [129, 130, 131]. Both American and European guidelines advocate lifelong SAPT in patients without an indication for OAC, with DAPT reserved for those at elevated ischemic risk [132, 133]. The optimal regimen following successful TAVI remains uncertain, and ongoing trials aim to clarify best practices.

For patients with concurrent indication for OAC, the addition of APT must be individualized and carefully considered. Given that these patients are often elderly and at HBR, monotherapy with OAC is typically preferred. However, in those with recent ACS or PCI, dual therapy with OAC and SAPT for 1 to 6 months may be warranted. An individualized approach remains central to optimizing post-TAVI outcomes [126].

#### 7.1.2 Mitral and Tricuspid Valve Interventions

Transcatheter edge-to-edge repair has become an essential option for patients with severe symptomatic MR and TR who are unsuitable for surgical repair. It employs devices that approximate the valve leaflets to reduce regurgitation and improve hemodynamic function. In the absence of specific guidelines, antithrombotic therapy after TEER is guided by protocols from clinical trials and tailored to patient characteristics [134, 135, 136, 137]. A commonly adopted regimen consists of aspirin and clopidogrel for 1–6 months, followed by life-long aspirin. For patients with AF and those requiring lifelong OAC, maintaining therapy with VKA and targeting an international normalized ratio (INR) of 2.5 is the current standard of care [126, 138].

Transcatheter mitral valve replacement (TMVR) is emerging as a viable option for patients with complex mitral valve pathology who are ineligible for surgery or TEER. TMVR has demonstrated effectiveness in managing degenerated bioprostheses, failed annuloplasty rings, and mitral annular calcification in high risk patients [139]. Antithrombotic strategies generally mirror those for surgical bioprosthesis, with VKA recommended for the first 3 months post-implantation [140]. For transcatheter tricuspid valve replacement (TTVR), a 6-month course of VKA is typically used [141]. Long-term APT post-TMVR and TTVR remain under debate. While American guidelines support lifelong aspirin in patients without an OAC indication, European guidelines recommend aspirin only when other indications for APT exist [132, 140]. Long-term management should be personalized based on thrombotic and bleeding risk assessments and any additional indications for APT.

### 7.2 Occlusion of the Patent Foramen Ovale/Atrial Septal Defect/Ventricular Septal Defect

PFO and ASD are among the most common congenital heart defects. Device-based closure of these defects is a widely used intervention to prevent paradoxical embolism and reduce stroke risk. Post-procedural antithrombotic therapy is essential to mitigate the risk of device-related thrombosis and embolization, which occurs in approximately 2%–3% of cases [142]. As closure techniques for ASD and VSD resemble those for PFO, APT strategies for these defects are generally extrapolated from PFO data [143, 144, 145]. DAPT for 1 to 6 months, followed by lifelong aspirin, is the recommended regimen after device closure of PFO, ASD or VSD [126, 137].

### 7.3 Left Atrial Appendage Occlusion

For patients with AF and elevated thrombotic risk, OAC with VKA or DOAC remains the mainstay of stroke prevention. However, LAA occlusion provides an important alternative for those with HBR or contraindications to long-term OAC. Antithrombotic therapy post-LAA occlusion is crucial to prevent thrombus formation on the device and reduce embolic risk. Device-related thrombus rates range from 4% to 17.6%, highlighting patient heterogeneity and variability in closure devices, antithrombotic protocols, and study outcomes [126, 146].

Standard trial protocols recommend 45 days of VKA plus aspirin, followed by 6 months of DAPT in the absence of significant peri-device leaks, lifelong aspirin thereafter [147, 148]. In clinical practice, regimens vary significantly. DOAC, at full or reduced doses, has been explored as an alternative to VKA [149]. Observational studies suggest that APT alone may be sufficient without increasing thrombotic or stroke risk [150, 151]. A meta-analysis of HBR patients undergoing LAA occlusion found that SAPT was comparable with DAPT regarding stroke, device-related thrombus, and major bleeding outcomes [152]. In the absence of robust randomized control data, therapy decisions should be individualized based on clinical profile and risk assessment [153, 154].

In practice, a DAPT regimen for 1 to 6 months, preferably until adequate sealing of LAA, is considered appropriate, followed by long-term SAPT. For patients with high thrombotic and acceptable bleeding risk, aspirin combined with DOAC for 45 days, followed by 6 months of DAPT, may be considered [126, 155].

## 8. Conclusion

Antiplatelet therapy remains a cornerstone in the management of heart disease, particularly for preventing ischemic complications in patients with CAD undergoing PCI. Advances in antiplatelet agents over the years have significantly enhanced our ability to reduce thrombotic risk effectively. However, the benefits of intensified platelet inhibition are counterbalanced by an increased risk of bleeding, highlighting the importance of a personalized approach to therapy. Careful assessment of ischemic versus bleeding risks, along with individual patient factors, such as prior bleeding history, remains essential to tailoring treatment strategies.

Emerging approaches, including shortened durations of DAPT, P2Y_12_ inhibitor monotherapy, and de-escalation protocols, seek to reduce bleeding risk without compromising efficacy. Conversely, intensified regimens, such as prolonged DAPT or dual pathway inhibition, may be appropriate for patients with elevated ischemic risk and low bleeding susceptibility. These personalized strategies represent a move toward precision medicine in antithrombotic management [156]. Innovative tools like genotyping and PFT hold promise for guiding therapy by predicting individual drug responsiveness, though their clinical utility is still being established. The growing emphasis on patient-centered care is refining APT by customizing agent selection, dosing, and duration based on genetic profiles, procedural considerations, and individualized risk stratification.

Antiplatelet therapy is a dynamic and continuously evolving field. A forward-looking perspective, grounded in a thorough understanding of current evidence, is essential for advancing patient-centered care and optimizing outcomes across a wide range of clinical scenarios.
